# Loss of *Dec1* inhibits alcohol-induced hepatic lipid accumulation and circadian rhythm disorder

**DOI:** 10.1186/s12860-023-00497-y

**Published:** 2024-01-02

**Authors:** Fuyuki Sato, Ujjal K. Bhawal, Kosuke Oikawa, Yasuteru Muragaki

**Affiliations:** 1 Department of Diagnostic Pathology, Shizuoka Cancer Center, Sunto-gun, 411-8777 Japan; 2 Department of Pathology, Wakayama Medical University School of Medicine, Wakayama, 641- 8509 Japan; 3 Research Institute of Oral Science, Nihon University School of Dentistry at Matsudo, Chiba, 271-8587 Japan; 4 Center for Global Health Research , Saveetha Medical College and Hospitals , Saveetha Institute of Medical and Technical Sciences, Saveetha University, Chennai, 600077 India

**Keywords:** Dec1, Alcohol, Immunohistochemistry, AMPK, PPARs

## Abstract

Chronic alcohol exposure increases liver damage such as lipid accumulation and hepatitis, resulting in hepatic cirrhosis. Chronic alcohol intake is known to disturb circadian rhythms in humans and animals. DEC1, a basic helix-loop-helix transcription factor, plays an important role in the circadian rhythm, inflammation, immune responses, and tumor progression. We have previously shown that *Dec1* deficiency inhibits stresses such as periodontal inflammation and perivascular fibrosis of the heart. However, the significance of *Dec1* deficiency in chronic alcohol consumption remains unclear. In the present study, we investigated whether the biological stress caused by chronic alcohol intake is inhibited in *Dec1* knockout mice. We treated control and *Dec1* knockout mice for three months by providing free access to 10% alcohol. The *Dec1* knockout mice consumed more alcohol than control mice, however, we observed severe hepatic lipid accumulation and circadian rhythm disturbance in control mice. In contrast, *Dec1* knockout mice exhibited little effect on these outcomes. We also investigated the expression of peroxisome proliferator-activated receptors (PPARs) and AMP-activated protein kinase (AMPK), which are involved in the regulation of fatty acid metabolism. Immunohistochemical analysis revealed increases of phosphorylation AMPK and PPARa but decreases PPARg in *Dec1* knockout mice compared to that in control mice. This indicates a molecular basis for the inhibition of hepatic lipid accumulation in alcohol-treated *Dec1* knockout mice. These results suggest a novel function of *Dec1* in alcohol-induced hepatic lipid accumulation and circadian rhythm disorders.

**Supplementary Information:**

The online version contains supplementary material available at 10.1186/s12860-023-00497-y.

## Background

Chronic alcohol intake causes hepatitis, fibrosis, and fatty liver, and promotes hepatocellular carcinoma [1–3]. Several molecular mechanisms involving hepatic lipid metabolism have been reported [1, 2]. AMP-activated protein kinase (AMPK) is a serine/threonine protein kinase whose activation requires phosphorylation at Thr172 [4]. AMPK regulates glucose and lipid metabolism [1, 5]. Chronic alcohol exposure inhibits AMPK activity, leading to steatosis and liver injury [6–8]. Treatment with an AMPK activator, 5-aminoimidazole-4-carboxamide-1-b-D-ribofuranoside (AICAR), inhibits alcohol-induced fatty lipid accumulation in the liver [6, 9]. In contrast, peroxisome proliferator-activated receptors (PPARs) are nuclear receptors associated with fatty liver diseases [1]. PPARa is abundantly expressed in the liver, heart, kidney, and gastrointestinal tract; PPARb/d are ubiquitously expressed, but dominantly expressed in the skeletal muscle, skin, and adipose tissue; and PPARg is highly expressed in the adipose tissue and has low expression in the liver [10]. However, increased PPARg expression has been observed in fatty liver and chronic alcohol intake [11–13].

The circadian rhythm is an important factor to maintain health. Disturbances in the circadian rhythm can induce various diseases, such as sleep disorders, cancer, and metabolic syndrome [14–17]. The circadian rhythm is predominantly regulated by clock genes, such as *CLOCK*, *brain and muscle Arnt-like protein (BMAL) 1*, *period (PER) 1/2/3*, *cryptochrome (CRY) 1/2*, and *differentiated embryonic chondrocyte gene (DEC) 1/2 (also known as BHLHE40/41)* [16, 17]. Additionally, chronic alcohol treatment disrupts circadian rhythms and locomotor activity in mice [18, 19]. Furthermore, alcohol consumption in humans increases the amplitude of clock genes such as *CLOCK, BMAL1, PER1, CRY1*, and *CRY2* [20]. These reports suggest that chronic alcohol exposure disturbs circadian rhythms by inducing abnormalities in the clock genes. DEC1 is a basic helix-loop-helix transcription factor that regulates tissue differentiation, cell proliferation, inflammation, tumor progression, and the circadian rhythm [16, 17, 21–23]. DEC1 negatively regulates phosphorylated AMPK (pAMPK) through liver kinase B1 (LKB1) by binding to the E-box site of the LKB1 promoter [24]. DEC1 also negatively regulates PPARg by binding to the C/EBP site of its promoter [25, 26]. However, the pathogenesis of the alcohol-induced damage caused by *Dec1* remains unclear. Thus, in this study, we examined the role of *Dec1* in chronic alcohol exposure.

## Materials and methods

### Animals

Animal experiments were conducted according to protocols approved by the Animal Care and Use Committee of Wakayama Medical University (approval number 660). For the experiments, mice were instantaneously sacrificed by cervical spine dislocation, and the liver tissue was removed, fixed in 4% paraformaldehyde solution, and embedded in paraffin. Whole *Dec1* deletion in *Dec1* knockout (KO) mice with a C57BL/6 background was performed as previously described [21, 27]. Eight-to-nine-week-old C57BL/6 wild-type (WT) and *Dec1* KO female mice were housed under 12:12 h light:dark (lights on at 8:00 a.m., ZT0; lights off at 8:00 p.m., ZT12) conditions, as previously described [21, 27]. Ethanol was diluted with water to a final concentration of 10%. Mice were allowed free access to 10% ethanol for 3 months (WT, n = 9; *Dec1* KO, n = 9). The amount of alcohol consumed was calculated from the amount of alcohol remaining in the bottle every ten days. Statistical analyses were performed using Student’s *t-test*. Mouse livers were sampled at ZT2 and subjected to immunohistochemistry.

### Activity recording

Mouse locomotor activity was monitored using Supermex (Muromachi Kikai, Tokyo, Japan) (WT, n = 6; *Dec1* KO, n = 6), and data were recorded as previously described [22]. Activity tests were conducted for at least 40 days.

### Immunohistochemistry

Immunohistochemistry was performed using a Discovery Auto-Stainer with automated protocols (Ventana Medical Systems, Inc., Tucson, AZ, USA; Roche, Mannheim, Germany; Ventana Discovery XT, NexES version 10.6 software, DAB-Map kit 760 − 124), as previously described [22, 28]. The pAMPK antibody (rabbit monoclonal, 40H9, 2535, and 1:300) was purchased from Cell Signaling Technology, Inc. (MA, USA). Total AMPK antibody (mouse monoclonal, D-6, sc-74,461, 1:50), PPARg antibody (mouse monoclonal, E-8, sc-7273, 1:50), and PPARa antibody (mouse monoclonal, H-2, sc-398,394, 1:50) were purchased from Santa Cruz Biotechnology Inc (TX, USA). We have previously clarified the specific immunostaining for pAMPK and total AMPK antibodies in the liver and heart [24]. We performed negative control staining and confirmed that no primary antibodies were detected in tissues without secondary antibodies.

### Statistical analyses

Statistical analyses were performed using a Student’s *t-test* in Excel software.

## Results

### *Dec1* KO mice consumed more alcohol than the WT mice

**Fig. 1:**
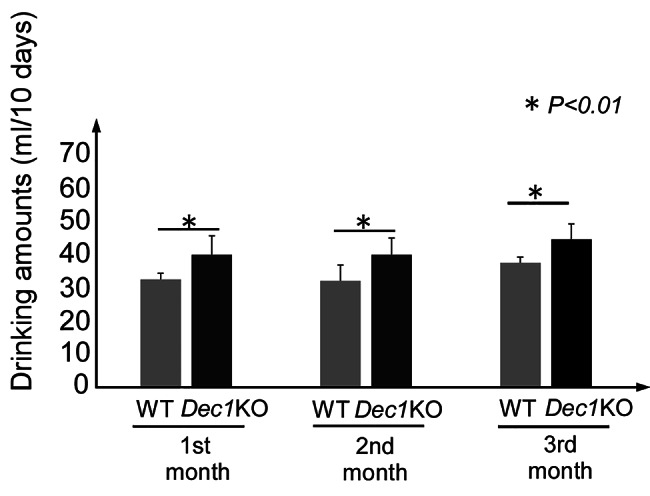
*Dec*1 KO mice consumed more alcohol than WT mice. Drinking quantities of 10% alcohol in *Dec1* KO and WT mice every month for 3 months. Drinking quantities in nine independent samples were evaluated by the liquid amounts remaining in the bottle. The raw data are shown in the Supplementary File. Each value represents the mean + SE (bars) of nine independent samples. 0-1 M: amount from start to 1 month, 1-2 M: amount from 1 to 2 month, 2-3 M: amount from 2 to 3 month. *P < 0.01, based on the t-test

We compared the amount of 10% alcohol consumed by *Dec1* KO and WT mice under free-access conditions. Subsequently, we calculated the amount of alcohol consumed every month for three months. The data demonstrated that *Dec1* KO mice consumed more alcohol than WT mice at all time points (Fig. 1).

### *Dec1* KO mice maintained circadian rhythm when consuming alcohol

**Fig. 2:**
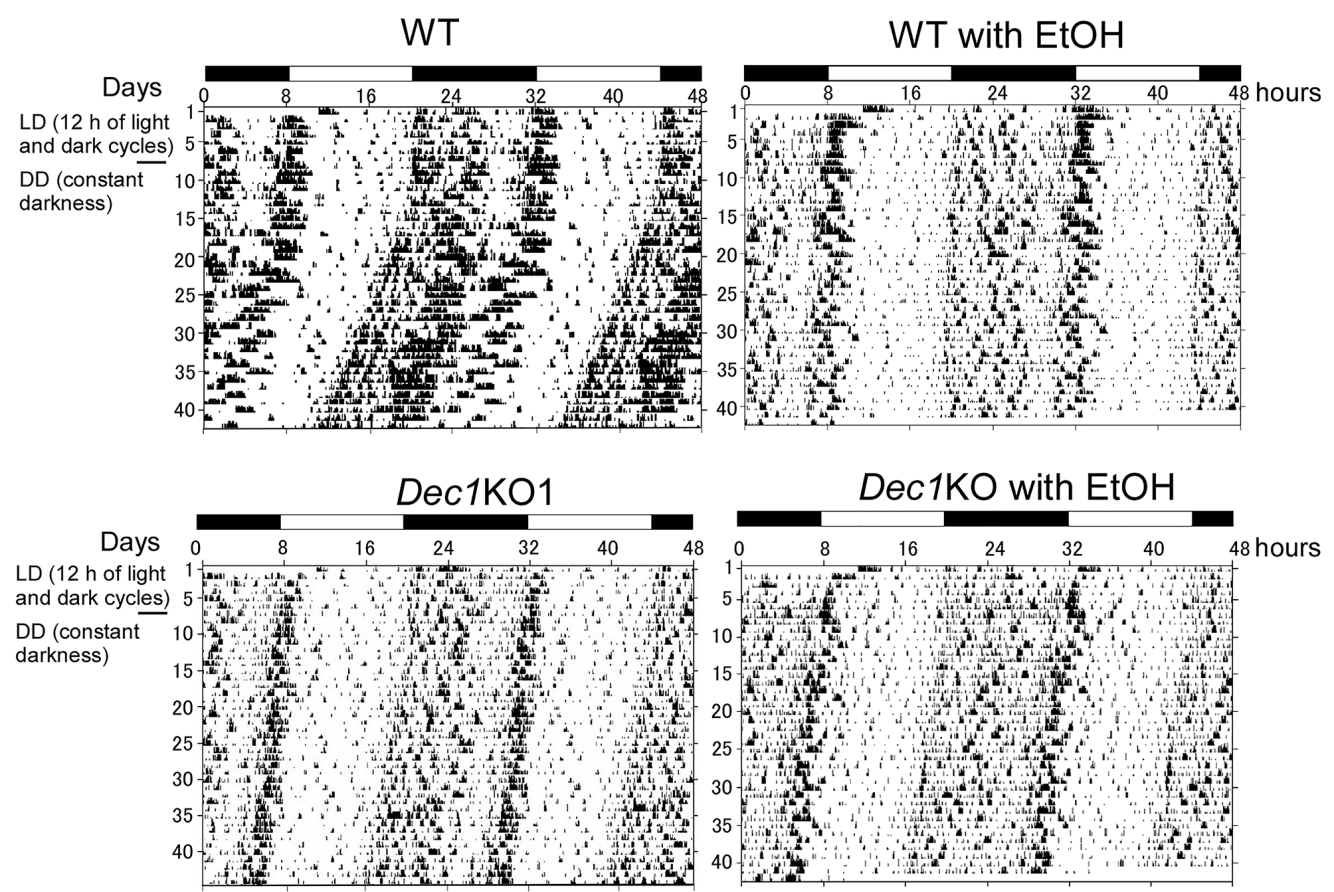
*Dec1* KO mice had little change in locomotor activity with 10% alcohol. Representative locomotor activity records of WT and *Dec1* KO mice with or without 10% alcohol. WT: WT mice without 10% alcohol, *Dec1* KO: *Dec1* KO mice without 10% alcohol, WT with 10% EtOH: WT mice with 10% alcohol, *Dec1* KO with EtOH: *Dec1* KO mice with 10% alcohol. LD: 12 h of light and dark cycles, DD: constant darkness. Black squares indicate the periods of darkness. DD started on day 7

Next, we examined the spontaneous locomotor activity of *Dec1* KO and WT mice with or without 10% alcohol consumption using SuperMex. The mice were fed under light/dark (LD) conditions for six days and then shifted to constant dark (DD) conditions. As shown in Fig. 2, the locomotor activity of the WT mice that did not consume alcohol exhibited a phase shift after shifting to the DD condition. Alcohol consumption in WT mice decreased locomotor activity and did not result in a phase shift after DD (Fig. 2). In contrast, the locomotor activities of *Dec1* KO mice were lower than those of WT mice without alcohol consumption and were minimally affected by alcohol consumption.

### Lipid accumulation induced by drinking 10% alcohol was inhibited in *Dec1* KO mice via pAMPK, PPARa, and PPARg

**Fig. 3:**
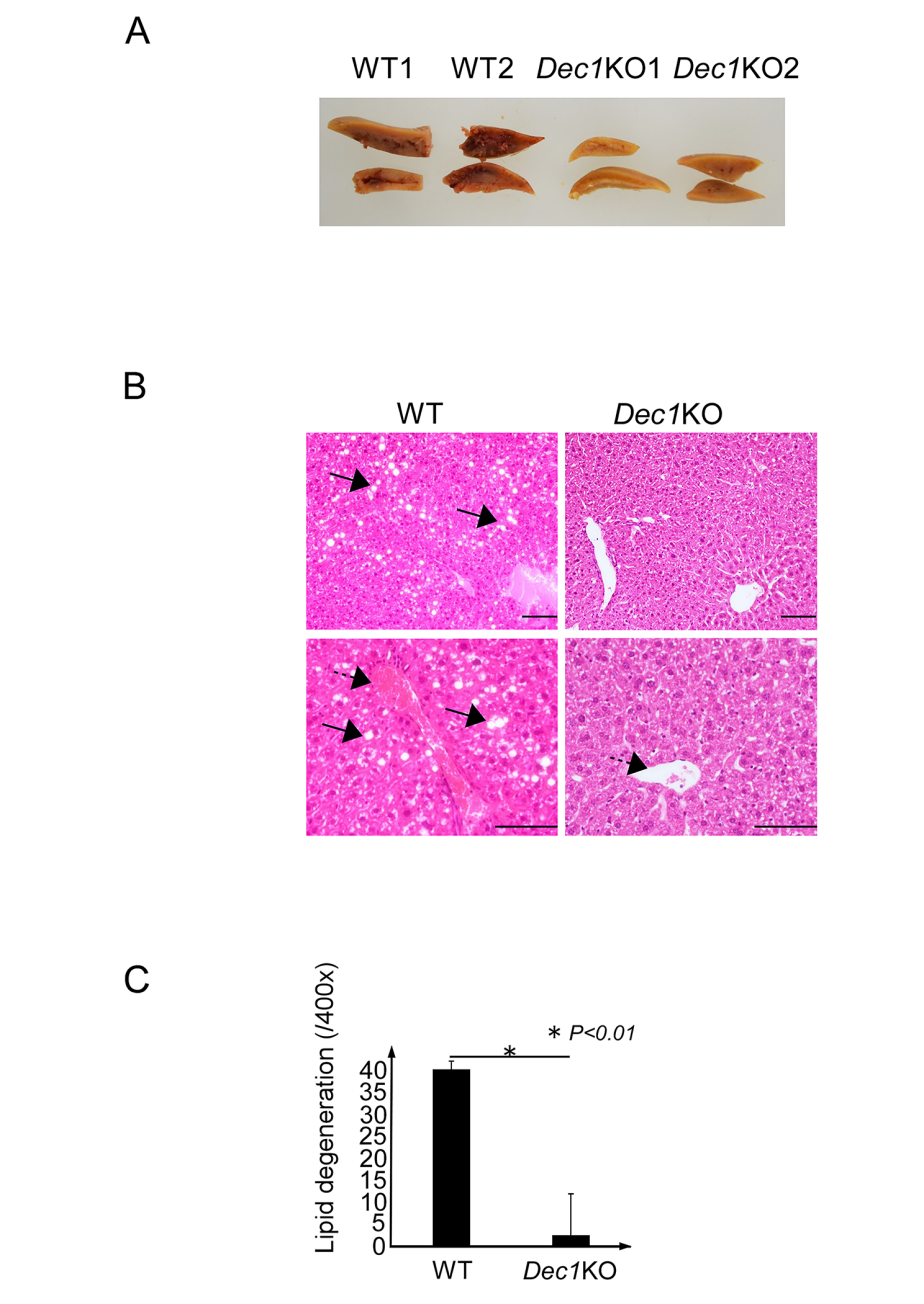
*Dec1* KO mice had inhibited fatty acid degeneration induced by 10% alcohol. **(A)** Macroscopic features of two independent WT (WT1 and WT2) and *Dec1* KO (*Dec1* KO1 and *Dec1* KO2) livers after 10% alcohol consumption. WT mice show severe hepatic congestion, but *Dec1* KO mice show little change. **(B)** Microscopy revealed severe lipid degeneration and congestion in the livers of WT mice, whereas *Dec1* KO mice were minimally affected. Top panel, 200 x magnification. Bottom panel, 400 x magnification. Arrows show lipid degeneration. Dotted arrows show central vein. Scale bars, 100 µM. **(C)** Average numbers of lipid degeneration locations in three independent samples. Lipid degeneration around the central vein was counted in ten microscopic fields under 400 x magnification. *P < 0.01, based on the t-test. The raw data are shown in the Supplementary File

**Fig. 4:**
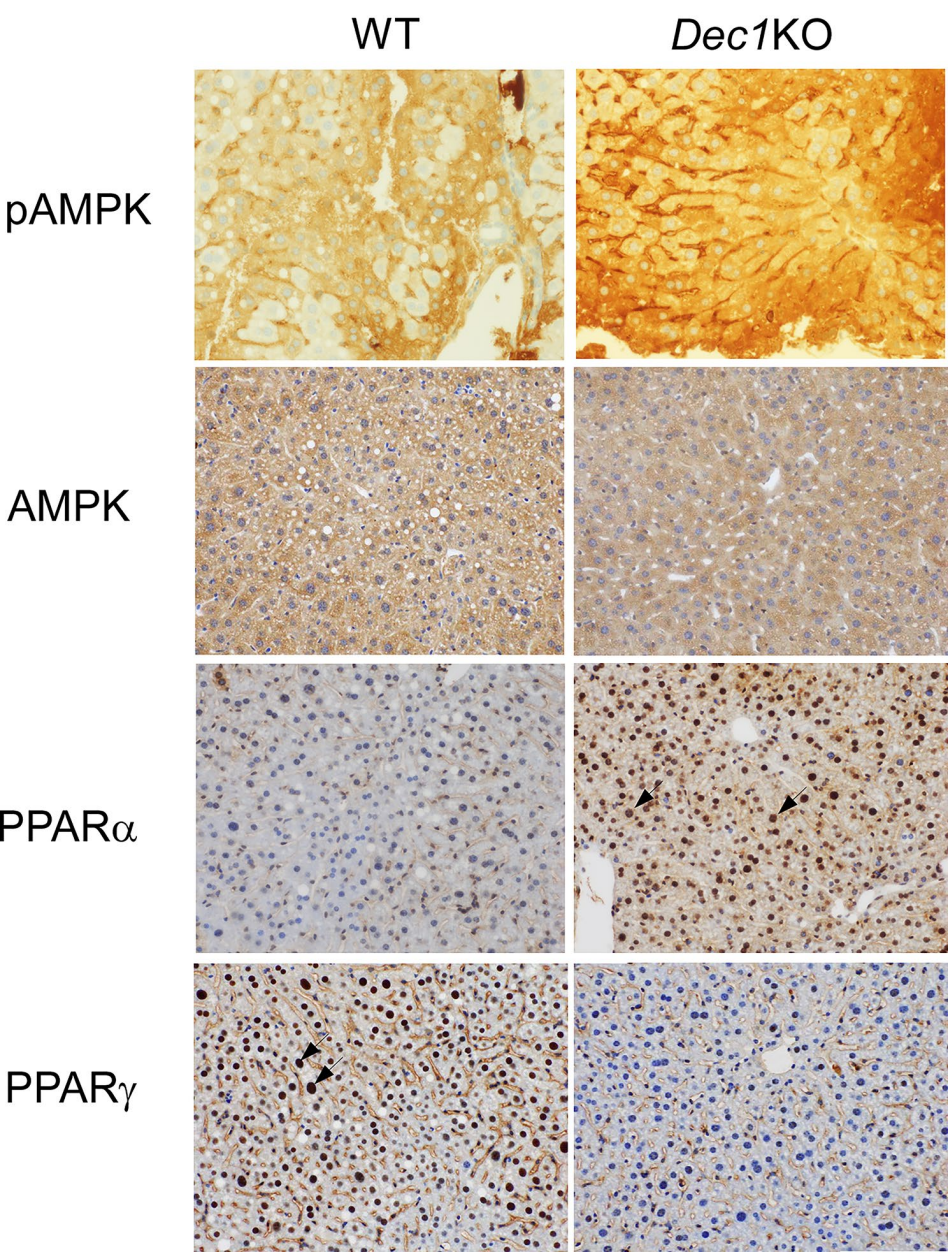
pAMPK, PPARa and PPARg immunoreactivities in the liver of WT and *Dec1* KO mice with 10% alcohol consumption. *Dec1* KO mice exhibited higher phosphorylated AMPK (pAMPK), but had little impact on total AMPK. *Dec1* KO mice had higher nuclear expression of PPARa compared to that in WT mice; PPARg expression in *Dec1* KO mice was lower. 400 x magnification. Scale bars, 100 µM

Next, we analyzed the livers of WT and *Dec1* KO mice treated with 10% alcohol for three months. The livers of WT mice showed severe congestion and fat accumulation in response to alcohol consumption, whereas those of *Dec1 KO* mice showed little change (Fig. 3A and B). Lipid generation in *Dec1* KO mice treated with 10% alcohol was approximately 14-fold lower than that in WT mice (Fig. 3C). The livers were quickly extracted and fixed in 4% paraformaldehyde solution. We observed significant lipid degeneration in hepatic cells of WT mice. In contrast, all *Dec1* KO mice showed little effect. We believe that degeneration was no affected by sample preparation. Furthermore, we investigated the expression of pAMPK, PPARa and PPARg, which are involved in the regulation of fatty acid metabolism, by immunohistochemistry. We observed increased expression of pAMPK in *Dec1* KO mice, but the expression levels of total AMPK remained almost the same between WT and *Dec1* KO mice (Fig. 4). Moreover, the nuclear amounts of PPARa were higher and PPARg were lower in *Dec1* KO mice compared to those in WT mice.

## Discussion

We have previously shown that *Dec1* KO inhibits periodontal inflammation and perivascular fibrosis in the oral mucosa and heart [21, 27, 29]. Thus, *Dec1* KO may provide protection against various stressors. However, it was unclear whether *Dec1* KO inhibits the influence of stress associated with chronic alcohol exposure on drinking. We revealed that chronic alcohol intake induced a disturbance in the circadian rhythm and lipid accumulation in WT mice, but these abnormal phenotypes did not occur in *Dec1* KO mice. Surprisingly, *Dec1* KO mice consumed more alcohol than WT mice. These results suggest that the abnormalities do not depend on the amount of alcohol consumed. We propose three hypotheses regarding the mechanism underlying the improvement in lipid accumulation in *Dec1* KO mice. First, *Dec1* deficiency functionally protected against stress induced by 10% alcohol intake. Ren et al. showed that cardiac-specific *Dec1* knockdown inhibits atrial inflammation and fibrosis [30]. Therefore, *Dec1* deficiency may inhibit of chronic alcohol consumption. Second, *Dec1* deficiency may eliminate ingested alcohol via urine. Third, *Dec1* deficiency may increase the absorption of ingested alcohol. We have previously reported that *Dec1* deficiency restores age-related metabolic imbalance by inducing the expression of liver fibroblast growth factor 21 (FGF21) [29]. Therefore, *Dec1* deficiency might protect against or ameliorate stress.

The induction of pAMPK has been shown to inhibit liver damage by alcohol [1, 5, 6, 8]. We have previously demonstrated that DEC1 negatively regulates pAMPK via LKB1 [24]. Thus, *Dec1* deficiency may suppress lipid accumulation and liver damage by inducing the expression of LKB1 and pAMPK.

It has also been reported that chronic alcohol exposure increases PPARg expression, but decreases PPARa [5, 11–13]. Other researchers showed that Dec1 negatively regulates PPARg [25, 26]. However, our results revealed that *Dec1*deficiency inhibited PPARg expression. Dec1 function differs for various types of stress [16]. We speculate that Dec1 promotes PPARg expression under chronic alcohol exposure, although detailed mechanisms are unknown. Dec1 may down-regulate PPARa but up-regulate PPARg under chronic exposure, suggesting that Dec1 may regulate target PPARs through different mechanisms. Further studies are needed to elucidate the role of *Dec1* deficiency in alcohol absorption and elimination. No clear changes were observed in food intake, body weight, or fat amounts in WT and *Dec1* KO with or without chronic alcohol intake. Many previous studies showed that only chronic alcohol intake affected lipid metabolism and circadian rhythm [3, 18, 19, 31, 32]. Our results agree with these findings, and we believe that lipid degeneration and circadian rhythm disorders depend on chronic alcohol intake but not on excessive food intake. Oil-Red O staining is useful method for determining hepatic lipid accumulation, although researchers must prepare frozen sections using raw tissues. We plan to use this method in the future.

Disturbance of the circadian rhythm induces abnormalities in the expression of clock genes, sleep, and metabolism, resulting in depression, dementia, metabolic syndrome, type2 diabetes, and cancer [17, 22, 33–36]. We showed that chronic alcohol intake disturbed the circadian rhythm of WT mice, but had little effect on *Dec1* KO mice. As previously mentioned, circadian disorder in *Dec1* KO mice may be inhibited by liver absorption and elimination of alcohol. Further studies are required to elucidate the detailed molecular mechanisms by which *Dec1* deficiency inhibits circadian disorders under stressful conditions.

## Conclusions

We conclude that *Dec1* deficiency inhibits lipid accumulation and circadian disorders caused by chronic alcohol consumption. These findings may contribute to the development of novel treatment strategies for chronic alcohol-induced damage.

### Electronic supplementary material

Below is the link to the electronic supplementary material.

Supplementary Material 1

Supplementary Material 2

## Data Availability

All the data analyzed in this study are included in the article. Raw data for the quantification of alcohol consumption and lipid degeneration are available in the Supplementary file.

## Abbreviations

Dec1: Differentiated embryonic chondrocyte gene 1
H&E: Hematoxylin and eosin
AMPK: AMP-activated protein kinase
